# Long-term results of revision total hip arthroplasty with a cemented femoral component

**DOI:** 10.1007/s00402-018-3023-9

**Published:** 2018-08-11

**Authors:** Armin Pallaver, Lukas Zwicky, Lilianna Bolliger, Hans Bösebeck, Isabella Manzoni, Sabine Schädelin, Peter E. Ochsner, Martin Clauss

**Affiliations:** 1 Clinic for Orthopedic and Trauma Surgery, Kantonsspital Baselland, Rheinstrasse 26, 4410 Liestal, Switzerland; 2 Heraeus Medical GmbH, Wehrheim, Germany; 3 Clinical Trial Unit, University Hospital of Basel, Basel, Switzerland

**Keywords:** Cemented revision THA, French paradox, Straight stem

## Abstract

**Introduction:**

In revision total hip arthroplasty (THA), the cancellous bone is normally completely removed out of the femoral canal during stem extraction. This situation is comparable to primary THA following the shape-closed concept, with some authors advocating to remove the metaphyseal cancellous bone to enhance press-fit stability (“French paradox”). The aim of this study was to investigate the long-term outcome, regarding survival and radiological results, of a cemented straight stem when used for revision THA and to compare these results to the results of the same stem in primary THA.

**Materials and methods:**

178 stem revisions performed between 01/1994 and 08/2008 using the Virtec straight stem were included. The cumulative incidence for re-revision was calculated using a competing risk model. Risk factors for re-revision of the stem were analyzed using an absolute risk regression model. Radiographs analyzed for osteolysis, debonding and subsidence had a minimum follow-up of 10 years.

**Results:**

The cumulative incidence for re-revision due to aseptic loosening of the stem was 5.5% (95% CI, 2.9–10.2%) at 10 years. Aseptic loosening was associated with younger age, larger defect size and larger stem size. After a minimum 10-year follow-up, osteolysis was seen in 39 of 80 revision THA. Compared to the results in primary THA, the survival in revision THA with the same implant was inferior.

**Conclusions:**

Cemented straight stems used for revision THA showed excellent long-term results regarding survivorship and radiological outcome. This stem therefore offers a valuable and cost-effective option in revision THA.

## Introduction

Revision total hip arthroplasty (THA) is a continuously increasing procedure in orthopedic surgery [1, 2]. Like in primary THA, stability of components can be achieved with or without bone cement. Using uncemented revision implants on the femoral side has become more and more popular. They show excellent long-term results even in patients who have poor femoral bone stock [3]. However, a potential disadvantage of uncemented revision stems is the partial weight bearing allowed in the immediate postoperative period to prevent excessive stem subsidence, especially when using a transfemoral approach [4, 5]. This disadvantage of uncemented stems can be bypassed when using cemented femoral revision stems, where immediate postoperative weight bearing is always possible.

In the literature, long-term results of cemented femoral revision using impaction bone grafting have shown excellent long-term survival [6, 7]. However, this technique is demanding and the periprosthetic fracture rate is reported to be high [8]. Furthermore, it is expensive to keep a bone bank or to buy lyophilized allograft bone chips.

In primary-cemented THA, there are two main basic theoretical design principles for stem fixation: shape-closed/composite beam, and force-closed/loaded taper [9]. For force-closed fixation, an undersized implant is inserted and surrounded by a complete, thick cement mantle while for shape-closed fixation, the largest implant possible is inserted leaving only a thin and/or incomplete cement mantle. The latter technique is also known as “French paradox” [10]. In this concept, the resulting press-fit to the cortical bone enhances the fixation of the stem [11]. Some authors even advocate the removal of cancellous bone using a curette to even further enhance the press-fit stability of the stem [12] with excellent long-term results [13]. This situation is comparable to the situation found in revision THA, where cancellous bone as well as all granulation tissue is normally completely removed out of the femoral canal during stem extraction. Up to now, only sparse knowledge exists on using a shape-closed stem in cemented femoral revision arthroplasty.

The aim of the present study was (1) to investigate the long-term outcome regarding survivorship and radiological results of a cemented straight stem using the shape-closed concept in revision THA and (2) to compare these results to data of the same stem used in primary THA.

## Patients and methods

This is an observational, single-center study which is based on prospectively collected data of a consecutive series of patients.

### Implants and patients

The Virtec straight stem (Zimmer, Winterthur, Switzerland) is a variant of the Müller straight stem. The stem is double-tapered, has an oval cross section and is made of CoNiCrMo [14]. The design rationale was, analogous to the Kerboull stem [12], to provide better filling of the proximal femur when compared to the original Müller straight stem [14]. Various sizes in standard and lateral versions are available. In the first period of this study, the PF stem (Cedior, Montbéliard, France) with the same shape, however, manufactured out of stainless steel, was used. As results did not differ between the two implants, no differentiation was made for further evaluation (data not shown).

All 172 patients (178 hips) who underwent revision with the PF or Virtec straight stem between 01/1994 and 08/2006 were included. Ethics committee approval was obtained prior to study commencement. The mean age (standard deviation) of the patients at the time of the surgery was 68.4 (9.3) years (range 36–90 years); 126 (71%) cases were male. Indication for revision was aseptic loosening in 137 cases (77%), periprosthetic joint infection (PJI) in 32 cases (18%), malposition of the stem in 6 cases (3.4%), and a broken implant in 3 cases (1.7%). The type of explanted stems are listed and femoral defects prior to replantation classified according to Della Valle and Paprosky [15] shown in Table 1. 116 cases received their primary implantation in our hospital, 62 cases were referred to us from another hospital. A total of 19 cases had at least one previous revision.

**Table 1:** Type of explanted stems and preoperative defect sizes

Stems	*n*	%	Defect size (type)					
			I	II	IIIa	IIIb	IV	Missing
Müller-type straight (Ti)	82	46.1	14	28	31	2	–	7
Müller-type straight (CoCr)	44	24.7	14	14	11	1	–	4
Other cemented	31	17.4	8	10	8	1	–	4
Uncemented	19	10.7	10	5	3	–	–	1
Uncemented revision	2	1.1	1	1	–	–	–	–

A revision of the acetabular component was performed in 82 out of 178 cases. In 61 cases, a cemented Mueller acetabular reinforcement ring was implanted, in 11 cases, a non-cemented SL-cup, and in 10 cases, a Burch-Schneider anti-protrusio cage (all Zimmer, Winterthur, Switzerland).

### Surgical technique

**Fig. 1:**
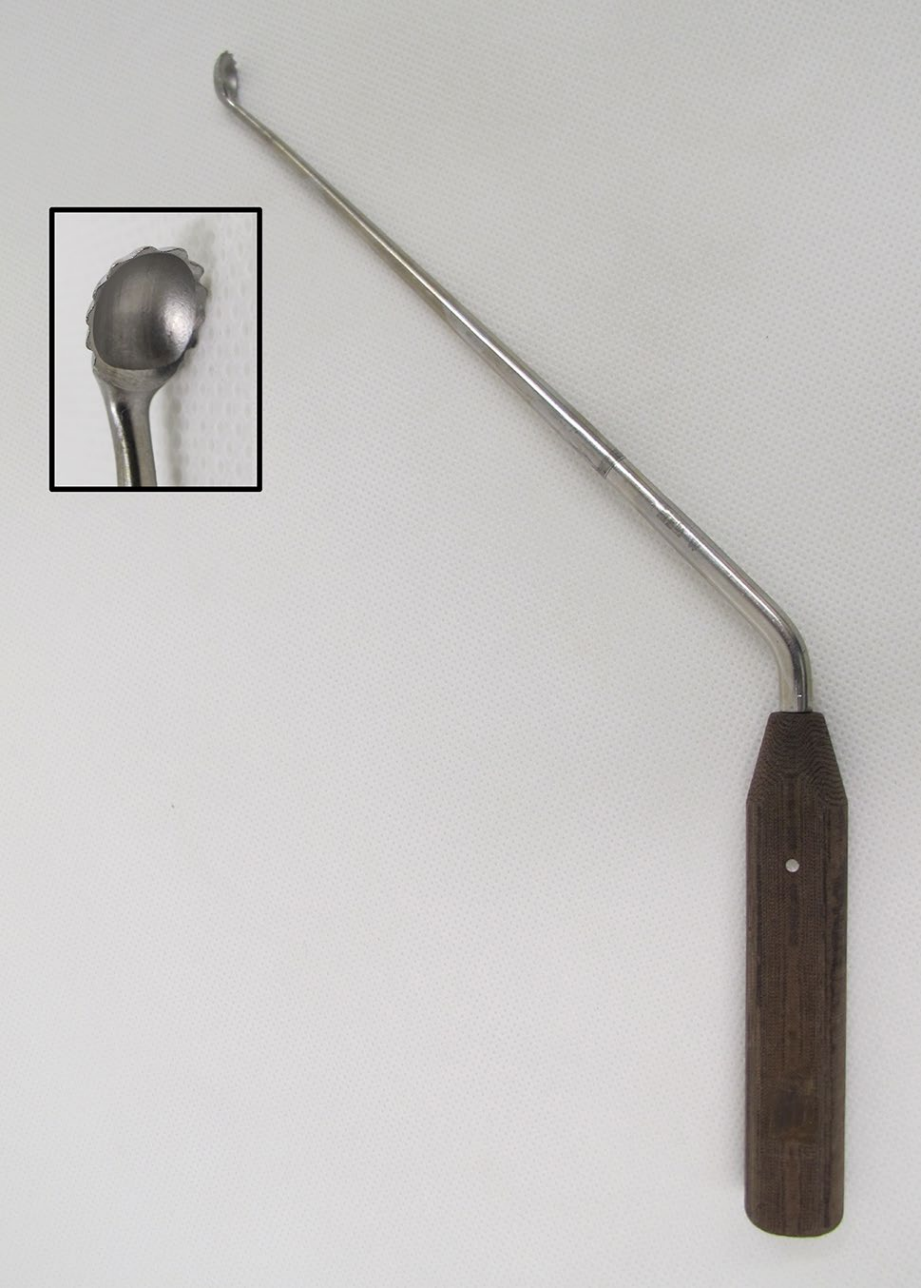
A specially designed curette was used to remove all granulation tissue

Every revision surgery was standardized using a lateral transgluteal approach with the patient in a supine position. After stem removal, the medullary canal was cleaned using drills and chisels to remove all cement remnants; granulation tissue was completely removed using specially designed curettes (Fig. 1). Thereafter, the medullary canal was repeatedly rinsed with a 0.2% polyhexanide solution (Lavasept^®^, B. Braun, Melsungen, Germany) using a bulb syringe. Subsequently, the largest implant fitting into the medullary canal was implanted, aiming for a primary press-fit fixation in the anterior–posterior (ap) plane (Table 2). Implants were cemented line-to-line with the final broach [16]. All stems where implanted with a second-generation cementing technique (no vacuum mixing, distal plug, retrograde filling) using high-viscosity Palacos R + G (Heraeus, Hanau, Germany) cement [17]. In case of septic loosening, patients were treated according to our established algorithm either with a 1-stage (14 cases) or 2-stage revision (18 cases) [18]. No additional antibiotics were added to the cement in any cases. The postoperative mobilization was the same for all patients with initial full weight bearing as tolerated starting the first postoperative day.

**Table 2:** Implant specifications of implanted stems

	Revision THA		Primary THA	
	*n*	%	*n*	%
Size				
5	3	1.7	42	14.9
6	4	2.2	58	20.6
7	12	6.7	81	28.7
8	34	19.1	53	18.8
9	52	29.2	32	11.3
10	45	25.3	12	4.3
11	19	10.7	2	0.7
12	9	5.1	2	0.7
Offset				
Standard	45	25.3	72	25.5
Lateral	133	74.7	210	74.5

### Follow-up

Follow-up examinations were scheduled at 4 months, 1 year, 2 years, and 5 years, and all 5 years thereafter. Patients were rated as lost to follow-up when last contact (outpatient clinic or telephone call) was 5 years overdue. For radiological analysis, the first postoperative radiograph and the most recent radiograph of all unrevised patients with a minimum radiological follow-up of > 10 years or in case of a revision, the last radiograph prior to revision, were analyzed. Standardized AP radiographs were taken centered on the symphysis, showing the entire implant. Findings were stratified according to the Gruen zone system [19]. Osteolysis was defined as any newly developed progressive endosteal bone loss at the cement–bone interface with a diameter > 3 mm and categorized as either scallop- or bead-shaped [20]. Axial subsidence of the stem was measured as an increase of any radiolucency in the proximal cement in Gruen zone 1, created due to distal migration of the shoulder of the prosthesis. It was considered relevant if it was more than 2 mm [21]. Debonding was defined as present, if a radiolucent line at the prosthesis–cement interface, not visible on the first postoperative radiograph, was observed [22]. The stem was rated as being radiologically loose if circumferential radiolucency in all Gruen zones [23] and/or excessive subsidence of more than 10 mm [14] was present. The radiological analysis was carried out by one of the first authors at the end of the study; ambiguous findings were discussed with the senior investigator and agreed upon.

### Statistics

A survival analysis with death as a competing risk was performed with various endpoints: (1) aseptic loosening of the stem, (2) worst-case scenario with all cases lost to follow-up judged as aseptic loosening, and (3) re-revision of the stem and/or cup (including exchange of the liner) for any reason. Patients without any re-revision were censored at the date of last contact. For each endpoint, cumulative incidence functions were used. Furthermore, time to aseptic loosening of the stem was analyzed using an absolute risk regression model with death as a competing risk. The included variables were age, sex, preoperative defect, stem offset, and stem size.

A sensitivity analysis was performed using only the first hip in patients with bilateral stem revision. Since it resulted in similar estimates, no joint frailty model was estimated. Data of six patients with bilateral stem revisions were analyzed as independent.

A part of the results from a previously published study using the Virtec straight stem in primary THA (same stem, same bone cement) were compared to the results from the current study [14]. Cumulative incidence for aseptic loosening of the stem was compared according to Gray [24].

Continuous data were presented as mean (standard deviation) with range. Categorical data were presented as frequencies (percentages). The analysis was performed using a significance level of *α* = 0.05. R statistical package was used for all analyses.

## Results

Preoperative femoral defect types were distributed as followed: 47 hips (24.7%) with defect type 1, 58 hips (32.6%) with defect type II, 57 hips with defect type IIIa/b (32%) and no hips with defect type IV (Table 1).

Out of 178 hips there was no revision in 145 cases: a total of 74 patients (77 hips) had died after 9.1 (5.0) years (range 0.0–19.6 years) of causes unrelated to the THA without re-revision. 9 hips were lost to follow-up after 6.1 (4.4) years (range 1.6–14.0 years). The mean clinical follow-up was 9.3 (5.2) years (range 0–20.5 years). 80 hips had a more than 10-year radiographic follow-up (Fig. 3).

A total of 23 stems (12.9%) were re-revised: 15 for aseptic loosening, 2 after a periprosthetic femoral fracture and 6 due to PJI. Out of these six cases with PJI, two occurred after aseptic stem revision, one for persistent infection with coagulase negative staphylococci (CNS), and three had a further PJI with a new germ after previously being revised for PJI. In addition, there was one isolated cup revision during follow-up.

**Fig. 2:**
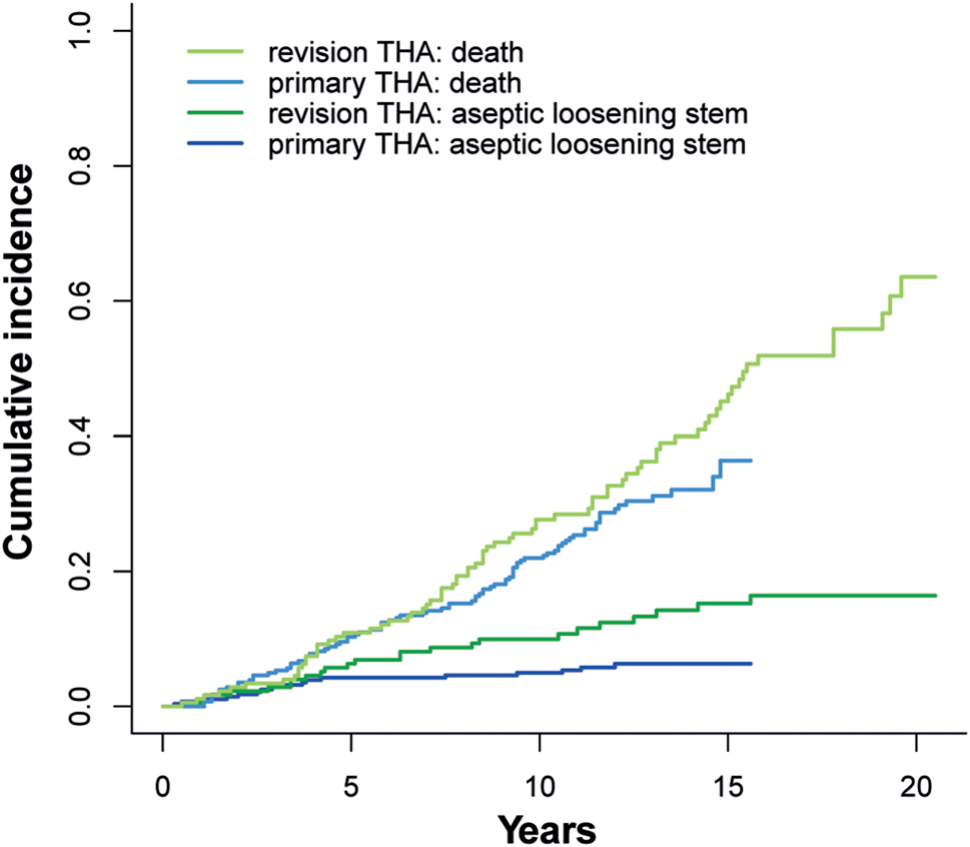
Cumulative incidence plot for aseptic loosening of the stem and death for primary THA and revision THA group

The cumulative incidence for aseptic loosening of the stem at 10 years was 5.5% [95% confidence interval (CI) 2.9–10.2%), whereas the cumulative incidence for death was 30.1% (95% CI 23.6–37.8%, Fig. 2). The cumulative incidence for aseptic loosening of the stem at 10 years assuming all patients which were lost to follow-up had been re-revised for aseptic loosening (worst-case scenario), was 9.9% (95% CI 6.3–15.4%).

The cumulative incidence for re-revision for any reason at 10 years was 9.9% (95% CI 6.3–15.5%).

The absolute risk ratios (ARR) for aseptic loosening of the stem are presented in Table 3. Aseptic loosening is associated with younger age (*p* = 0.02), larger defect size (*p* = 0.01/*p* = 0.07) and larger stem size (*p* < 0.001).

**Table 3:** Absolute risk ratio (ARR) with 95% confidence intervals (CI) for aseptic re-revision of the stem

	ARR	95% CI	*p* value
Age (years)	0.78	0.66–0.97	0.02
Sex (male vs. female)	26.93	0.52–1405.03	0.10
Stem offset (std vs. lat)	19.00	0.76–475.57	0.07
Stem size	7.43	2.00–27.65	< 0.001
Defect size (2 vs. 1)	3.74	1.46–9.55	0.01
Defect size (3 vs. 1)	1.54	0.96–2.49	0.07

**Fig. 3:**
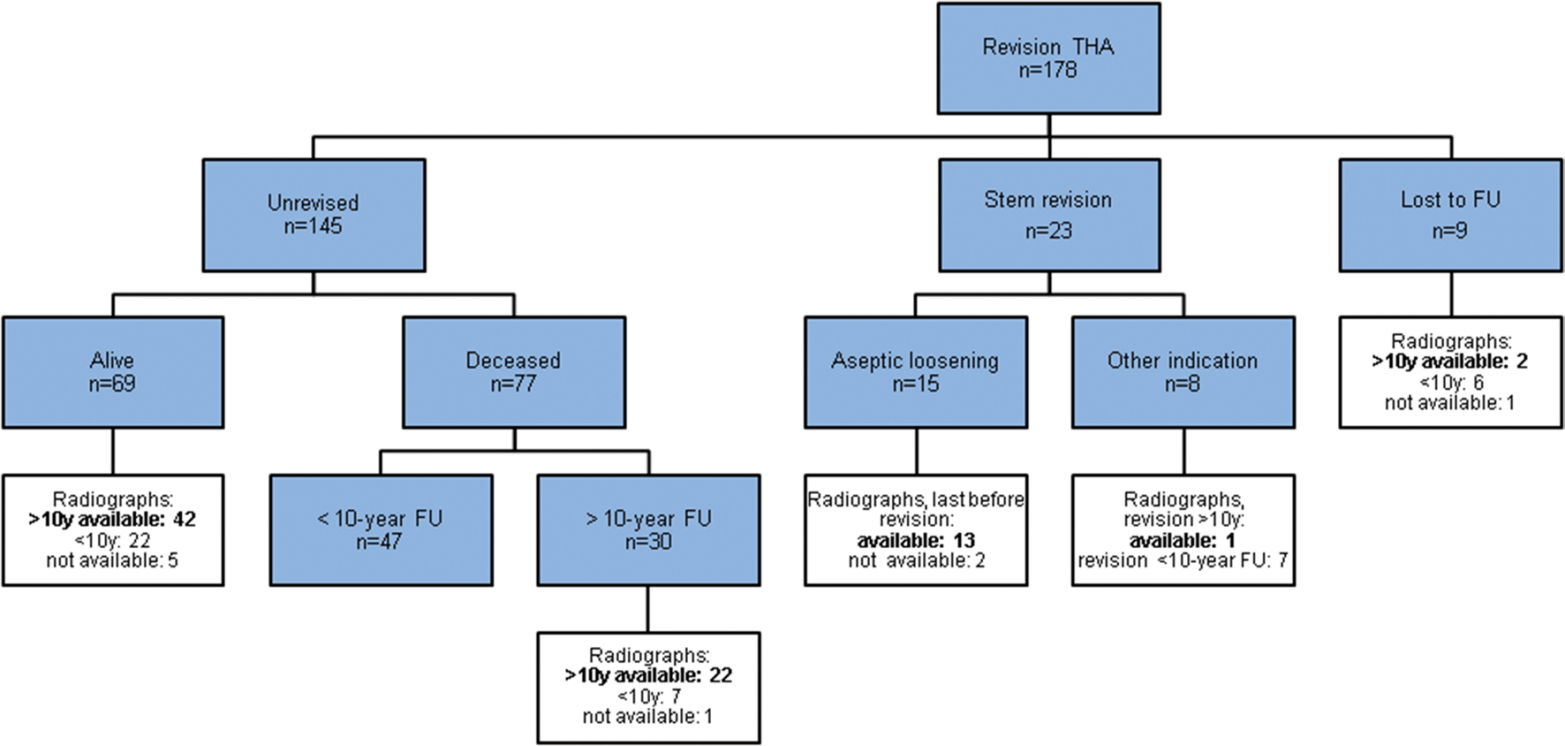
Survival status at the last follow-up and available radiographs for analysis (bold)

80 stems were eligible for a detailed radiological long-term analysis (Fig. 3). The average radiological follow-up for all unrevised stems was 12.9 (3.5) years (range 10–20.5 years) and 12.1 (4.2) years (range 0.7–20.5 years) for all hips, including the re-revised cases.

**Fig. 4:**
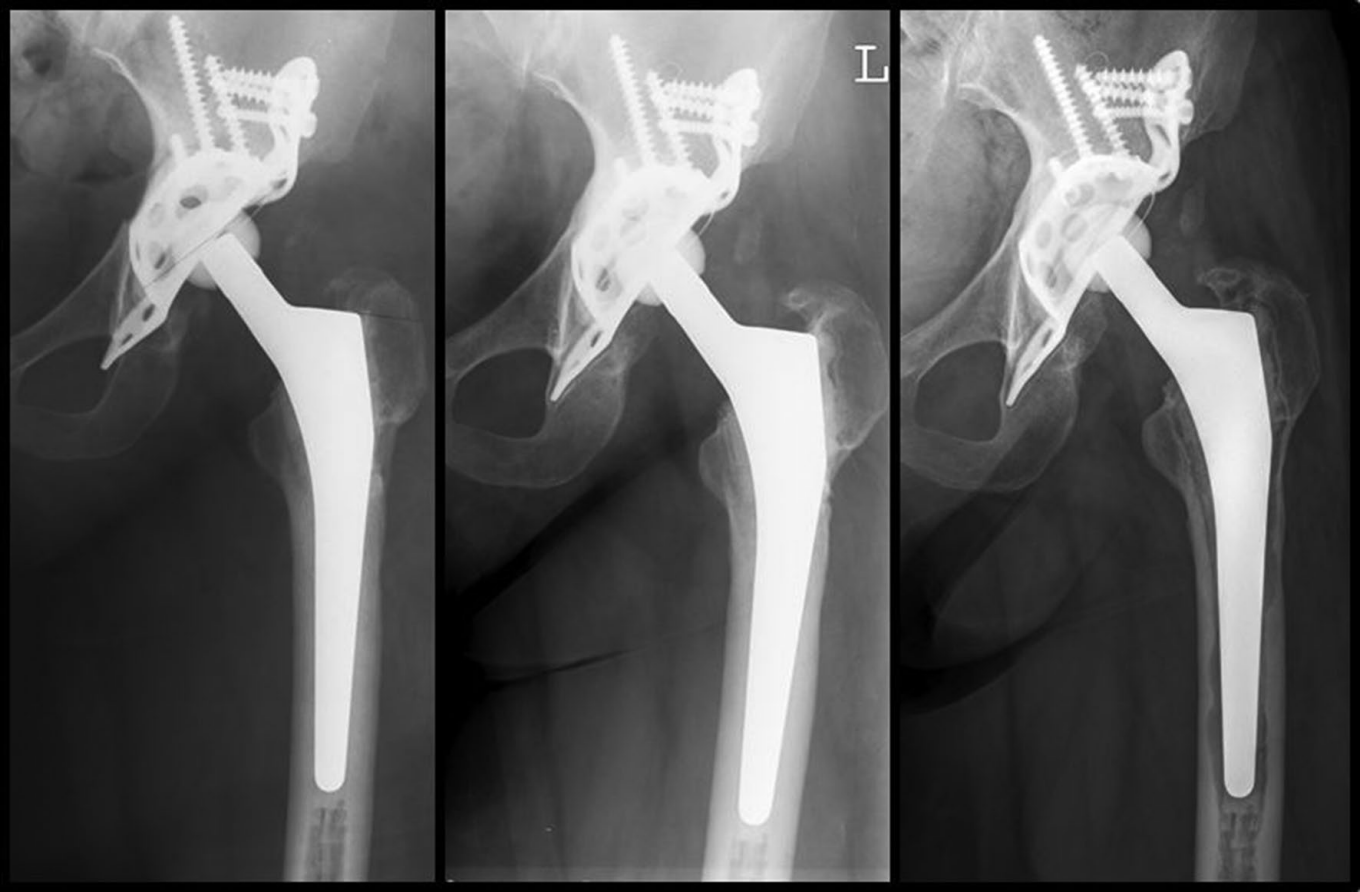
Cemented stem revision (Virtec stem and Burch-Schneider anti protrusion cage) of a 58-year-old woman, revision due to septic loosening. Preoperative defect Paprovsky grad 2. Radiographic follow-up postoperative (**a**), 10 years (**b**) and after 13 years (**c**) postoperative

Osteolysis was found in 39 of 80 stems (48.8%, Table 4; Fig. 4). 10 cases (12.5%) showed a circumferential radiolucency in all Gruen zones (of which 8 were re-revised). 13 cases (16.3%) showed a significant subsidence of more than 2 mm, median subsidence of these cases was 6 mm (range 3–10 mm). 5 out of 13 cases with subsidence were re-revised for aseptic loosening.

**Table 4:** Osteolysis

	Revision THA		Primary THA	
	*n*	%	*n*	%
Any	39	48.8	19	12.1
G1	30	37.5	3	1.9
G2	22	27.5	8	5.1
G3	17	21.3	4	2.5
G4	14	17.5	4	2.5
G5	17	21.3	6	3.8
G6	23	28.8	11	7.0
G7	23	28.8	10	6.4
G1–7	10	12.5	0	0.0

Debonding was rare and was only seen in two cases. No fracture of the cement mantle was observed.

Results were compared to a part of previously published data obtained using the same implant and bone cement but for primary THA (survival data of 268 patients; 282 hips) [14]. Age at the time of implantation, sex and BMI were comparable in both groups. The only difference between the groups was the stem size implanted. In the revision group, generally larger stems (sizes 11 and 12) were used.

For the primary THA group, the cumulative incidence for aseptic loosening of the stem at 10 years was 1.8% (95% CI 0.8–4.3%). This was significantly lower when compared to revision THA (*p* = 0.01). No significant difference could be found between the cumulative incidence for death at 10 years for the primary THA group [23.3% (95% CI: 19.1–29.2%)] and for the revision THA group (*p* = 0.58).

The cumulative incidence for revision for any reason at 10 years for primary THA was 5.0% (95% CI 3.0–8.2%).

## Discussion

The current study presents long-term results, in terms of survivorship and radiological outcome, for the cemented Virtec straight stem when used in revision THA. The present findings suggest excellent long-term results in revision THA when using the Virtec straight stem and shape-closed fixation technique.

The cumulative incidence for re-revision due to aseptic loosening after 10 years in our series was 5.5% (95% CI 2.9–10.2%). This falls into the lower range of previously published re-revision rates (8–22%) reported for cemented stem revisions [25–30].

The majority of the stems, which were revised, were cemented titanium Müller-type straight stems which have known detrimental effects on survival rates [31, 32]. Becoming aware of this problem, patients were closely followed up at our institution, thus allowing early diagnosis of loosening. This may explain the rather small defect sizes observed, with almost 60% of the patients having defect size I or II.

A high mortality indicates higher comorbidities, which is known as a general risk factor for revision in THA [33]. 74 patients (77 hips) died during the study period in the revision cohort, while only 14 stems were re-revised for aseptic loosening. Therefore, a cumulative incidence method was used to analyze revision rates [34–36]. Although the effect may not be clinically significant, the Kaplan–Meier estimates of implant failure are biased and would overestimate the risk of revision [37]. Anyhow it can be noted that the mean age of the patients at the time of the index surgery in the revision group was comparable to the mean age of the patients in the primary cohort. Therefore, the age of the patients in the revision THA group, at the time of the first implantation, was even younger when compared to the primary THA group.

The use of a cemented straight stem in revision THA is an extremely attractive method from an economic point of view [38]. Furthermore, it allows immediate full weight bearing without the risk of stem subsidence [39]. This may be of advantage for elderly, often frail patients, where longer partial weight bearing results in longer functional recovery time or is even impossible.

Risk factors for aseptic loosening of the Virtec stem in this study were young age, large defect size and large stem size. While young age is in accordance with the findings described by Clauss et al. [32] and Hallan et al. [40] for primary THA, the increased risk for revision of large stems is contradictory to results found in the literature. In our revision THA collective, larger stems were used when compared to our collective of primary THA, indicating that cavitary defects were present. In patients with huge cavitary defects, the pressure exerted on the cement mantle during stem insertion might be lower than with a stem which fills the medullary canal completely, resulting in a poorer press-fit in the AP plane. Furthermore, with an undersized implant (i.e., a complete cement mantle), the cement mantle may act differently and might not follow the principle of the “French paradox” [10].

**Fig. 5:**
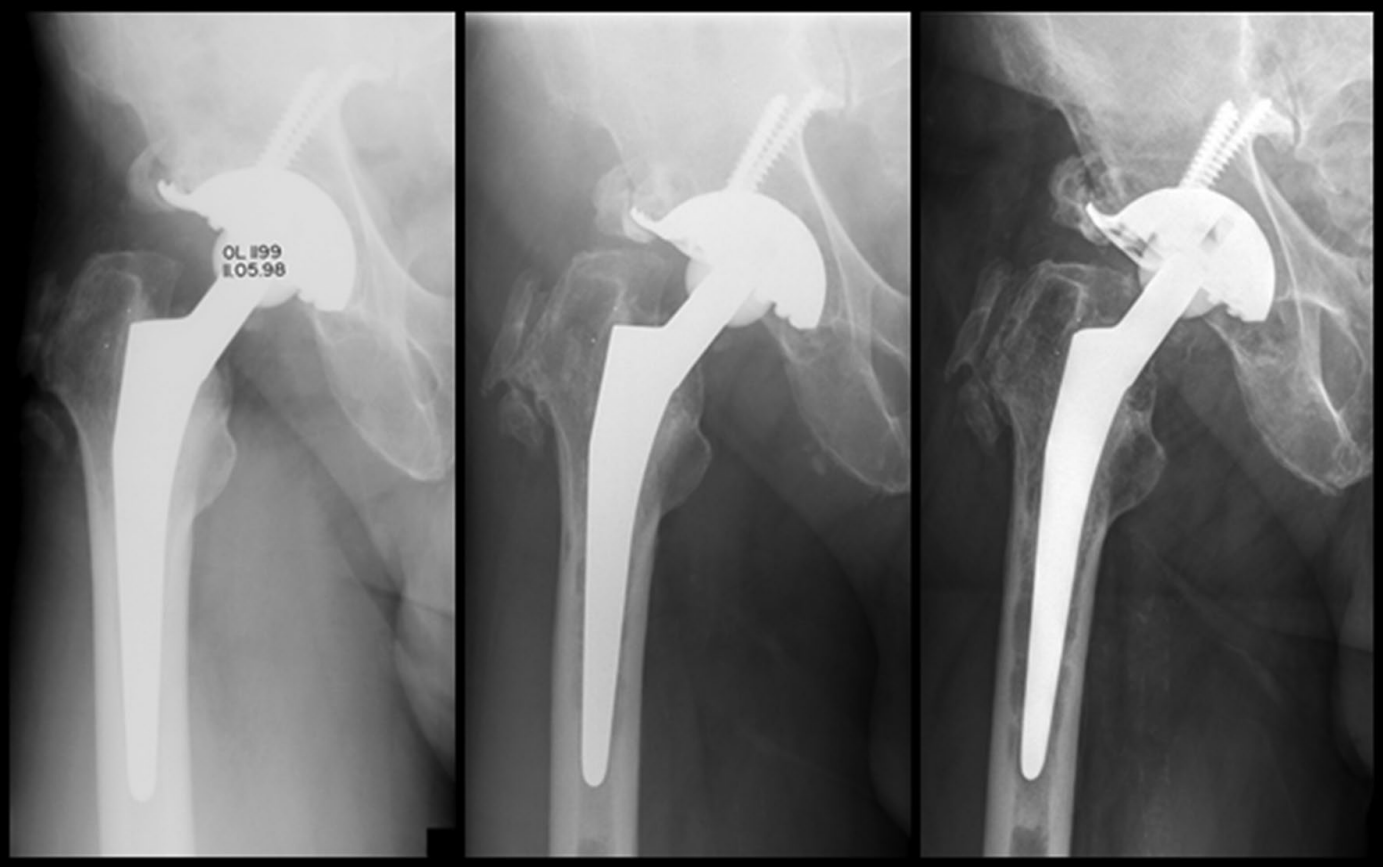
Cemented primary stem (Virtec stem and cementless SL-II cup) of a 86-year-old man, revision due to aseptic loosening. Radiographic follow-up 1 year (**a**), 10 years (**b**) and after 15 years (**c**) postoperative

Radiological changes around the stem were more frequent in the revision group compared to the series of primary THA. Osteolysis in any Gruen zone was found in 49% of all cases (39 of 80 stems) with a follow-up of more than 10 years in the revision THA group. However, only ten of these cases showed a circumferential radiolucency in all Gruen zones, eight of which were re-revised. Loosening, when rarely detected, always occurred at the cement–bone interface. This is normally observed in cemented stems with rougher surfaces were initially described by Gardiner et al. [41] Interestingly, the shape of the osteolysis was different in the revision THA group when compared to primary THA group. Osteolysis at the cement–bone interface in primary THA was more bead-shaped; in revision THA, osteolysis was more often like a scalloping radiolucent line without bead-shaped aspects (Figs. 4, 5). This finding may be due to the fact that during stem revision a meticulous debridement of all synovial membranes and remaining cancellous bone was achieved and a sound interdigitation between the cement and smooth inner cortex was not possible. This also explains the absence of debonding in the second interface (cement–stem) after a minimum follow-up of 10 years even with non-polished stems.

In conclusion, cemented straight stems, fixed according to the shape-closed principle, used in revision THA showed excellent long-term results regarding survivorship and radiological outcome. The mechanism of loosening might be different than in primary THA. Cemented straight stems offer a valuable and cost-effective option for revision THA, especially in the ever-growing number of geriatric patients requiring stem revision, where full weightbearing after surgery is favorable for functional recovery.
